# Seizures Do Not Affect Disability and Mortality Outcomes of Stroke: A Population-Based Study

**DOI:** 10.3390/jcm8112006

**Published:** 2019-11-17

**Authors:** Giovanni Merlino, Gian Luigi Gigli, Francesco Bax, Anna Serafini, Elisa Corazza, Mariarosaria Valente

**Affiliations:** 1 Clinical Neurology, University of Udine Medical School, 33100 Udine, Italy; gigli@uniud.it (G.L.G.); francescobax91@icloud.com (F.B.); mariarosaria.valente@asuiud.sanita.fvg.it (M.V.); 2 Neurology Unit, Department of Medicine, University of Udine, 33100 Udine, Italy; 3 Stroke Unit, Department of Neurosciences, Udine University Hospital, 33100 Udine, Italy; 4 DMIF, University of Udine, 33100 Udine, Italy; 5 Department of Neurology and Rehabilitation, University of Illinois at Chicago, Chicago, 60612 IL, USA; serafini.anna@gmail.com; 6 Department of Neurology, Hospital of Portogruaro, Portogruaro, 30026 Venice, Italy; eli.corazza@gmail.com

**Keywords:** acute stroke, post-stroke seizures, long-term outcome, disability, mortality

## Abstract

Although seizures are frequently seen after cerebrovascular accidents, their effects on long-term outcome in stroke patients are still unknown. Therefore, the aim of this study was to investigate the relationship between post-stroke seizures and the risk of long-term disability and mortality in stroke patients. This study is part of a larger population-based study. All patients were prospectively followed up by a face-to-face interview or a structured telephone interview. We enrolled 635 patients with first-ever stroke and without a history of seizures. Prevalence of ischemic stroke (IS) was 85.2%, while the remaining 14.8% of patients were affected by intracerebral hemorrhage (ICH). During the study period, 51 subjects (8%) developed post-stroke seizures. Patients with post-stroke seizures were younger, had a higher prevalence of ICH, had a more severe stroke at admission, were more likely to have an IS involving the total anterior circulation, and were more likely to have a lobar ICH than patients without seizures. Moreover, subjects with seizures had more frequently hemorrhagic transformation after IS and cortical strokes. At 24 months, the risk of disability in patients with seizures was almost twice than in those without seizures. However, the negative effect of seizures disappeared in multivariate analysis. Kaplan-Meier survival curves at 12 years were not significantly different between patients with and without post-stroke seizures. Using the Cox multivariate analysis, age, NIHSS at admission, and pre-stroke mRS were independently associated with all-cause long-term mortality. In our sample, seizures did not impair long-term outcome in patients affected by cerebrovascular accidents. The not significant, slight difference in favor of a better survival for patients with seizures may be attributed to the slight age difference between the two groups.

## 1. Introduction

Seizures are frequently seen after cerebrovascular accidents. Acute symptomatic or early seizures (ES) affect between 3% and 6% of all stroke patients [1,2,3,4,5,6], whereas unprovoked or late seizures (LS) have been reported in 10 to 12% of stroke patients [7,8].

Although determinants and correlates of ES and LS after stroke have been largely investigated [1,6,9,10,11,12,13,14,15,16,17], the effect of seizures on long-term outcome in stroke patients is still unknown. In fact, previous studies focused their interest on short-term mortality [2,12,18,19,20,21,22], and only a few of them reported results on disability that was assessed only at discharge [13,18,19,21,22].

Recently, Claessens et al. conducted a retrospective study to explore a possible association between post-stroke seizures and long-term mortality. After correction for possible confounding variables, the authors concluded that seizures were not significantly related to mortality risk [23]. Conversely, in the prospective *Future* study, Arntz et al. showed that post-stroke seizures negatively affect long-term disability and mortality [24,25]. Differences in the population study might explain these conflicting findings. In particular, Claessens et al. included only patients with intracerebral hemorrhage (ICH), whereas Arntz et al. included only young patients, aged 18 to 50 years, after transient ischemic attack (TIA), ischemic stroke (IS) or ICH [23,24,25]. Bearing in mind that ICH accounts for almost 15% of all strokes and that cerebrovascular accidents are largely more common in subjects older than 50 years, these results cannot be directly generalizable to all stroke patients.

Therefore, the aim of this study was to investigate the relationship between post-stroke seizures and the risk of long-term disability and mortality in patients affected by cerebrovascular accidents.

## 2. Methods

### 2.1. Study Population

This survey is part of a larger population-based study on the incidence and outcome of cerebrovascular disease in the district of Udine, Friuli-Venezia-Giulia region, Italy [26,27]. In short, this study comprises all cases of incident or recurrent TIA and strokes occurring in the Udine district between 1 April 2007 and 31 March 2009. Previous results on cumulative incidence and short-term mortality (at one month and 24 months) of post-stroke seizures have been published [6]. For the present study we included patients with first-ever stroke and without a history of seizures. According to the World Health Organization definition, stroke was defined as acute symptoms and/or signs of focal disturbance of cerebral function lasting >24 h or leading to death with no apparent cause other than that of vascular origin [28]. Exclusion criteria were TIA, traumatic hemorrhagic stroke, hemorrhage in known cerebral metastasis or primary brain tumor, cerebral venous sinus thrombosis, any subarachnoid hemorrhage or ICH due to known ruptured aneurysm, and retinal infarction.

The study was approved by our local Ethics Committee. Informed consent was obtained from the patients or their representatives.

### 2.2. Assessment of Post-Stroke Seizures

Seizures were defined according to International League Against Epilepsy (ILAE) criteria as paroxysmal disorders of the central nervous system, followed or not by loss of consciousness and/or with or without motor involvement [29]. The seizure type was described following the most recent classification of the ILAE [30]. Based on post-stroke seizures occurrence, patients were divided into SZR + (patients with post-stroke seizures) and SZR − (patients without post-stroke seizures) groups. The SZR + group comprised subjects with both ES and LS. Information on the use of antiepileptic drugs (AEDs) was recorded.

### 2.3. Data Collection

Baseline characteristics such as demographic data, vascular risk factor, and laboratory findings were collected. A CT or MRI brain scan was performed to distinguish IS from ICH and to identify stroke subtypes. IS subtypes were classified according to the TOAST (Trial of ORG 10172 in Acute Stroke Treatment) criteria (large artery atherosclerosis, cardioembolism, small-vessel occlusion, other determined etiology, and undetermined etiology) [31] and to the Oxfordshire Community Stroke Project (OCSP) criteria—total anterior circulation infarction (TACI), partial anterior circulation infarction (PACI), posterior circulation infarct (POCI), or lacunar infarct (LACI) [32]. Based on ICH location we distinguished lobar and deep ICH. Data on reperfusion therapy in patients with IS, hemorrhagic transformation after IS, and cortical location of cerebrovascular lesion were collected. However, the number of reperfused patients is very limited due to the fact that our center, at that time, was still at the beginning with this expertise. Stroke severity was determined with the National Institute of Health Stroke Scale (NIHSS) score at admission [33]. Functional outcome was assessed by means of the modified Rankin Scale (mRS) at admission based on pre-stroke disability, one month, six months, and 24 months after stroke [34]. The mRS score was dichotomized into favorable outcome (0–2) and poor outcome (3–6).

High blood pressure was defined as systolic pressure ≥140 mm Hg and/or diastolic pressure ≥90 mm Hg, and/or use of antihypertensive medication, and/or being told at least twice by a physician or other health professional that high blood pressure was the diagnosis. Atrial fibrillation was diagnosed if patient had atrial fibrillation in ECG recording before stroke and/or during hospitalization. Diabetes mellitus was defined as history of diabetes that was confirmed in medical records, and/or use of insulin/oral hypoglycemic agents, and/or random non-fasting blood glucose concentration ≥11.1 mmol/L. Hypercholesterolemia was defined as fasting total cholesterol serum level ≥5.18 mmol/L (200 mg/dL), and/or fasting low-density lipoprotein cholesterol serum level of ≥4.14 mmol/L (160 mg/dL), and/or use of lipid-lowering medications. Patients were defined as smokers if they were current smokers or they had stopped smoking <3 months before the index stroke.

The following abnormalities in laboratory parameters were collected: hypo- and hypernatremia, defined as sodium values <135 mmol/L and >145 mmol/L, respectively; hypo- and hyperkalemia, defined as potassium values <3.5 mmol/L and >5.1 mmol/L, respectively; and hypo- and hyperglycemia, defined as glucose values <3.9 mmol/L and >11.1 mmol/L, respectively.

### 2.4. Follow-Up

All patients were followed up by a face-to-face interview at one month, six months, and 24 months after stroke. At the follow-up, the occurrence of seizures, the beginning of AEDs, and the mRS were assessed. The next follow-up assessment was performed between January 2018 and December 2018 by means of a structured telephone interview to evaluate the occurrence of seizures. Before contacting the patient, the hospital records were reviewed for diagnosis of seizures. In case a patient had died, further information on the occurrence of seizures was retrieved from the general practitioner (GP). When patient or GP reported the occurrence of seizures, an experienced neurologist verified the diagnosis and retrieved information on AEDs.

### 2.5. Outcome Measures

The primary endpoints were

(1) Poor outcome at 24 months, as assessed by the mRS;
(2) All-cause long-term mortality. Information on the permanence in life was systematically recorded thanks to the use of death certificates.

### 2.6. Statistical Analysis

The two patient groups (SZR + versus SZR −) were compared regarding baseline characteristics, and rate of poor outcome at 24 months. Differences between the two groups were assessed by means of the chi-square test (Fisher’s exact test) for categorical variables and Student t test for independent samples when continuous variables had a normal distribution. The Mann–Whitney U test was used when continuous variables had an abnormal distribution and for ordinal variables. The association between post-stroke seizures and poor outcome was examined by binary logistic regression modeling, after adjusting for the other variables with a probability value <0.1 in univariate analysis. Long-term mortality was estimated with the Kaplan-Meier analysis for patients with and without post-stroke seizures. Independent predictors of mortality were detected by means of a Cox multivariate model that included age, sex, presence of post-stroke seizures, presence of IS or ICH, NIHSS at admission, and pre-stroke mRS, as covariates. Finally, we compared by means of the chi-square test the long-term disability and mortality between each group of patients, identified according to the specific type of post-stroke seizure, and the subjects without seizures. Data are displayed in tables as means and standard deviations (SD), if not otherwise specified. All probability values are two-tailed. A *p* value <0.05 was considered statistically significant. Statistical analysis was carried out using the IBMSPSS Statistics, Version 22.0 (IBM, Chicago, IL, USA).

## 3. Results

### 3.1. Study Population

**Figure 1:**
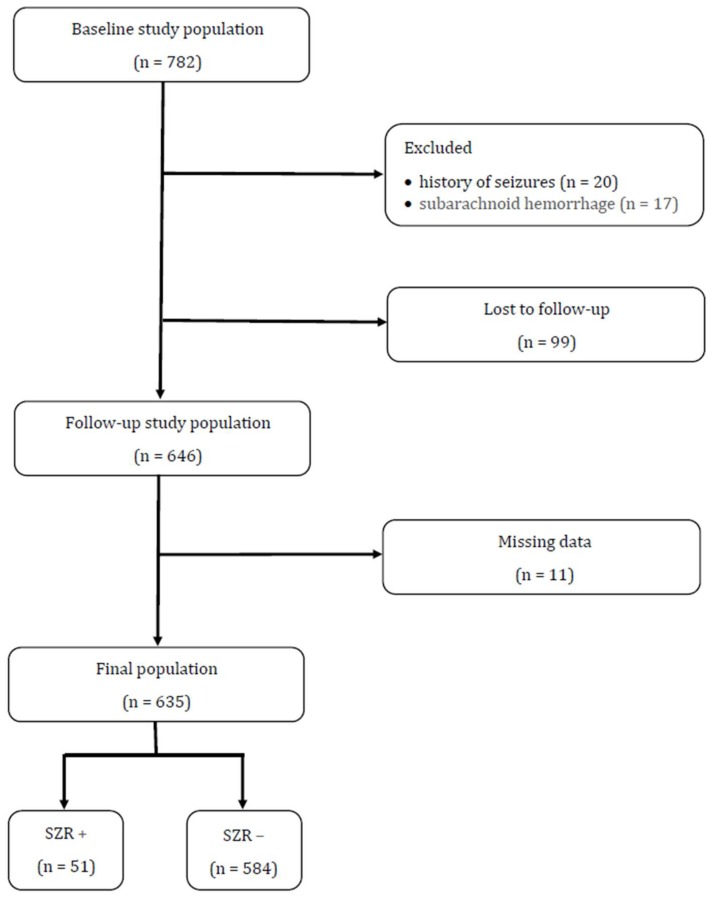
Study design. SZR +: patients with post-stroke seizures; SZR −: patients without post-stroke seizures.

Six hundred and thirty-five patients with first ever stroke entered the study (Figure 1). Prevalence of IS was 85.2% (n = 541), while the remaining 14.8% (n = 94) of patients was affected by ICH. Median NIHSS score at admission was 5 (IQR 2-15). One hundred and forty-one patients (22.2%) had a pre-stroke mRS >2. During the study period, 51 subjects (8%) developed post-stroke seizures, i.e., focal aware in 15 (29.4%), generalized tonic-clonic in 15 (29.4%), focal to bilateral tonic-clonic in 11 (21.6%), and focal impaired awareness in 10 (19.6%). AEDs were started in 21 patients (41.2%).

According to baseline characteristics, patients with post-stroke seizures differed significantly from those without seizures (see Table 1). They were younger, had a higher prevalence of ICH, had a more severe stroke at admission, were more likely to have an IS involving the total anterior circulation based on OCSP classification, and to have a lobar ICH. Moreover, subjects with seizures had more frequently hemorrhagic transformation after IS (SZR +: 31.4% versus SZR −: 13.7%, *p* = 0.005) and cortical strokes (SZR +: 52.9% versus SZR −: 32.5%, *p* = 0.003) than those without seizures. Although very limited in its numerosity, prevalence of rt-PA use for IS did not differ between patients with and without seizures (SZR +: 0% versus SZR −: 2.4%, *p* = 0.4). No patient was treated, at that time, with mechanical thrombectomy for IS.

### 3.2. Long-Term Functional Outcome

At 24 months, 419 patients (66%) had a poor outcome, 40 (9.5%) of whom had seizures. There were 216 patients (44%) without disability, and 11 (5.1%) of them had seizures. As reported in Table 2, several variables were associated with long-term disability in univariate analysis. In particular, the risk of poor functional outcome in patients with seizures was almost twice than in those without seizures. The negative effect of seizures disappeared in multivariate analysis that identified age, ICH, and NIHSS at admission as independent predictors of 24 months disability.

### 3.3. Long-Term Mortality

**Figure 2:**
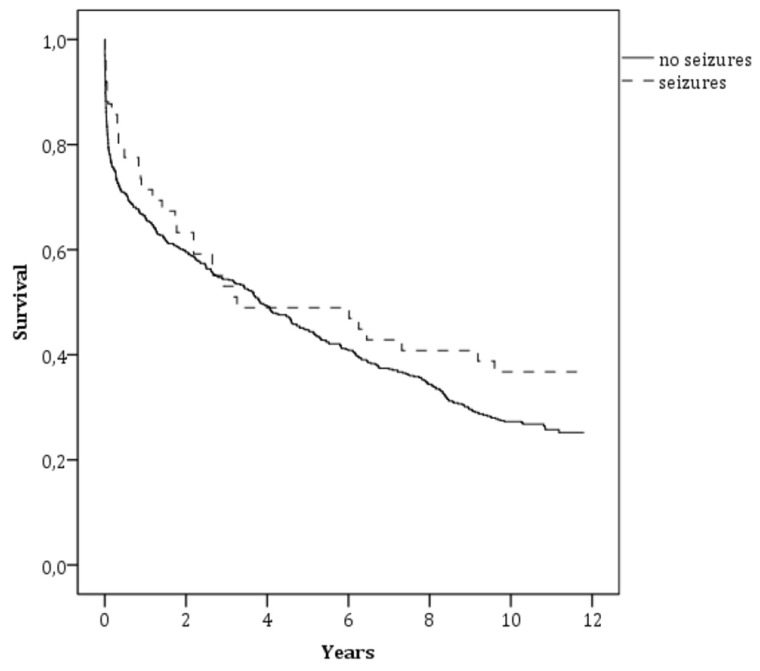
Survival curves for all-cause mortality according to post-stroke seizures.

Kaplan-Meier curves showed a no significantly higher survival rate for patients with post-stroke seizures compared to those without seizures (36.7% versus 26.2%; *p* = 0.2; Figure 2). Age, NIHSS at admission, and pre-stroke mRS were independently associated with all-cause long-term mortality at the Cox multivariate analysis (see Table 3).

### 3.4. Seizure Type and Long-Term Outcome

In Table 4, we compared prevalence of long-term disability and mortality between patients affected by each specific type of seizure and subjects without post-stroke seizures.

## 4. Discussion

This study shows that patients affected by post-stroke seizures do not have a long-term unfavorable outcome. In fact, rates of severe disability and mortality were comparable between stroke patients with and without seizures.

Previous hospital-based studies reported that the proportion of stroke patients experiencing seizures may vary from 1% to 25% [35]. Differently, information coming from population-based studies is more robust and repeatable. During the study period, we observed a prevalence of post-stroke seizures of 8%, which is perfectly in line with earlier data derived from studies performed at the community level [35].

In a previous study, we focused on identifying risk factors for post-stroke seizures. We reported that ICH, subarachnoid hemorrhage, stroke of undetermined origin and hyponatremia were independent predictors of ES, while LS risk factors were younger age and cortical location of stroke [6]. In the present study, post-stroke seizures were significantly associated with younger age, ICH and more severe stroke as assessed by NIHSS score at admission. Moreover, patients with total anterior IS, hemorrhagic transformation after IS, cortical strokes, and lobar ICH had a higher risk of seizures. In 1997, Arboix et al. reported that patients with seizures were significantly younger, and this result was replicated by Paolucci et al. in a cohort of 306 consecutive patients admitted to a rehabilitation hospital after their first ever stroke and later by Conrad et al. in 593 consecutive patients with different types of cerebrovascular events [12,36,37]. Similarly, in our sample the mean difference between subjects with and without seizures was almost five years. Given that the volume of cortical gray matter is higher in younger patients, a tentative explanation of this result was suggested to be due to the generally weaker epileptogenicity of older brains [36]. Several previous studies reported that stroke patients with ICH developed more frequently seizures than those with IS [14,38,39]. In a prospective multicenter study, Bladin et al. observed that ICH patients had an almost two-fold increase in risk of seizures after stroke [14]. Hemorrhagic transformation of IS has been found to increase the risk of seizures. In a prospective study on 368 patients with IS it was found that risk for developing seizures was almost 6-fold higher in subjects with hemorrhagic transformation [40]. This is usually attributed to the epileptogenicity of blood products (especially iron). In fact, free iron is a potent oxidizer that damages cell membranes due to creation of reactive oxygen species, peroxidation damage to cell membrane lipids, production of free radicals, and injury to oxidant-sensitive cellular enzymes, such as Na, K-ATPase [41]. The effects of hemoglobin and its degradation products (hemin and iron) on synaptic transmission was studied by Yip and Sastry in a rat model [42]. In addition, iron compounds, such as FeCl3, have been commonly used in animal models of epilepsy due to their highly epileptogenic effect when injected into the animal hippocampus and cortex [43,44]. It is known that seizures are more frequent in severe and disabling strokes [11,13]. Thus, our results regarding the association between seizure occurrence with stroke severity, based on NIHSS score at admission and presence of total anterior circulation infarct, agree with previous literature. Finally, we observed that cortical involvement was a risk factor for seizures in stroke patients. Several previous studies reported similar data [14,45,46].

Since stroke is, per se, the leading cause of disability, it is essential to identify further clinical conditions associated with the cerebrovascular accident that might affect functional outcome. To date, only few studies investigated the role of seizure in impairing disability after stroke [13,18,19,21,22,24]. In 1990, Kilpatrick et al. evaluated prospectively 1000 consecutive patients with stroke and TIA in order to establish incidence and consequence of ES. The authors did not show any association between seizures and worse functional outcome at discharge [18]. These results agree with the Dijon Stroke Registry, in which ES did not predict short-term disability after stroke [21]. In a different way, presence of ES predicted a better outcome at discharge in patients with acute stroke in the Copenhagen Stroke Study that adopted the Scandinavian Stroke Scale to measure stroke severity. The authors explained this surprisingly result by a relatively larger ischemic penumbra in patients who have an ES after a stroke [13]. The first study that used a validated tool for evaluating the effect of seizures on post-stroke disability was published in 2010. Patients with seizures had a greater disability having a mean mRS at discharge significantly higher than those without seizures (4.41 versus 3.15, *p* = 0.0001) [19]. Similarly, seizures during hospitalization after IS were associated with a higher morbidity at discharge, as measured by the mRS, in a recent large multicenter study [22]. To date, only the *Future* study investigated long-term (mean follow-up of 9.8 years) functional outcome in patients with and without post-stroke seizures. Arntz et al. reported that IS patients with epilepsy had a higher disability than those without it (mRS score >2: 27.5% versus 9.8%, *p* = 0.001). However, this result was not observed in patients with TIA and ICH [24]. Since this study was performed in young patients with cerebrovascular accidents, its consequences cannot be generalized. Our results, coming from a population-based study, should be considered more robust. During a 24-month follow-up, patients with post-stroke seizures had a poor functional outcome in unadjusted analysis, but this significant association was lost after multivariate analysis. Indeed, age, ICH, and NIHSS score at admission resulted to be the only independent predictors of long-term disability in our sample.

The relationship between post-stroke seizures and the risk of long-term mortality is largely misunderstood. In fact, previous studies focused on this topic had a short-term follow-up, i.e., one month or one year after stroke [2,13,18,19,20,21,22]. In 2015, Arntz et al. published new data of the *Future* study on post-stroke epilepsy and long-term mortality. The authors reported that the 20-year cumulative mortality for patients with and without post-stroke epilepsy was 56.5% and 32.6%, respectively. After adjustment for confounders post-stroke epilepsy remained significantly associated with mortality. As reported above, this study is limited because it enrolled only patients aged 18 to 50 years [25]. On the contrary, Claessens et al. observed that seizures after ICH were not significantly related to mortality risk after follow-up up to 10 years [23]. Our results agree with this last survey. In fact, long-term mortality risk did not differ in patients with and without post-stroke seizures. The not significant slight difference in favor of a better survival for patients with post-stroke seizures may be explained by the slight age difference between the two groups.

When we analyzed the effect of each specific type of seizure on long-term disability and mortality, we did not observe any significant association. However, there was a trend toward a lower mortality in patients with focal aware seizures and a higher disability in patients with focal to bilateral tonic-clonic seizures than in subjects without post-stroke seizures. Bearing in mind the small number of patients with seizures included in these analyses, we think that further larger studies focused on this topic are needed before any conclusion.

Strength of our study was the prospective design with data coming from a population-based study after one of the longest follow-ups (up to 12 years). There are also some limitations. Because of a long inclusion period, we cannot exclude that a recall bias might have influenced our results. Moreover, patients may underreport focal seizures with subtle semiology, thus misdiagnosis might have occurred. Finally, almost 12% of baseline study population was lost at follow-up and this loss might have minimally affected the results.

Despite these limitations, we believe that our results sufficiently demonstrate that seizures do not impair long-term disability and mortality in patients affected by cerebrovascular accidents.

## Figures and Tables

**Table 1:** Baseline characteristics.

	SZR + (n = 51)	SZR − (n = 584)	*p*
**Demographic Data and Baseline Clinical Characteristics**			
Age, years	72.3 ± 14.8	77.5 ± 11.5	0.003
Males, n (%)	21 (41.2)	276 (47.3)	0.4
*Stroke subtypes*		0.001	
IS, n (%)	35 (68.6)	506 (86.6)	
ICH, n (%)	16 (31.4)	78 (13.4)	
*IS subtypes based on TOAST classification*			0.08
Large artery atherosclerosis, n (%)	2 (5.7)	58 (11.5)	
Cardioembolism, n (%)	9 (25.7)	98 (19.4)	
Small-vessel occlusion, n (%)	1 (2.9)	82 (16.2)	
Other determined etiology, n (%)	2 (5.7)	9 (1.8)	
Undetermined etiology, n (%)	21 (60)	259 (51.2)	
*IS subtypes based on OCSP classification*		0.001	
TACI, n (%)	9 (25.7)	36 (7.1)	
PACI, n (%)	22 (62.9)	270 (53.4)	
POCI, n (%)	1 (2.9)	86 (17)	
LACI, n (%)	3 (8.6)	114 (22.5)	
*ICH localization*		0.01	
Lobar, n (%)	9 (56.3)	18 (23.1)	
Deep, n (%)	7 (43.8)	60 (76.9)	
NIHSS score at admission, median (IQR)	10 (4–17)	5 (2–14)	0.01
pre-stroke mRS, median (IQR)	0 (0-2)	0 (0-2)	0.2
**Vascular risk factors**			
Hypertension, n (%)	37 (72.5)	475 (81.3)	0.9
Atrial fibrillation, n (%)	17 (33.3)	195 (33.4)	0.4
Diabetes mellitus, n (%)	11 (21.6)	140 (24.0)	0.7
Hypercholesterolemia, n (%)	13 (25.5)	140 (24.0)	0.8
Smoking, n (%)	9 (17.6)	101 (17.3)	0.9
**Laboratory findings**			
*Sodium*		0.3	
Hyponatremia, n (%)	7 (13.7)	45 (7.7)	
Hypernatremia, n (%)	1 (2.0)	8 (1.4)	
*Potassium*		0.4	
Hypokalemia, n (%)	5 (9.8)	98 (16.8)	
Hyperkalemia, n (%)	4 (7.8)	37 (6.3)	
*Glucose*		0.2	
Hypoglycemia, n (%)	0 (0)	0 (0)	
Hyperglycemia, n (%)	37 (72.5)	370 (63.4)	

SZR +: patients with post-stroke seizures; SZR −: patients without post-stroke seizures; IS = ischemic stroke; ICH: intracranial hemorrhage; TOAST: Trial of ORG 10,172 in Acute Stroke Treatment; OCSP: Oxfordshire Community Stroke Project; TACI: total anterior circulation infarction; PACI: partial anterior circulation infarction; POCI: posterior circulation infarct; LACI: lacunar infarct; NIHSS: National Institute of Health Stroke Scale; mRS = modified Rankin Scale.

**Table 2:** Predictors of long-term disability.

Unadjusted Analysis		Multivariate Analysis *				
	OR	95% CI	*p*	OR	95% CI	*p*
Age	1.08	1.07–1.10	0.001	1.09	1.06–1.12	0.001
*Sex*						
Females	1.00					
Males	0.43	0.30–0.57	0.001	0.96	0.60–1.54	0.9
*Post–stroke seizures*						
No	1.00					
Yes	1.97	1.01–3.92	0.05	2.29	0.80–6–56	0.1
*Stroke subtypes*						
IS	1.00					
ICH	1.96	1.17–3.82	0.01	3.01	1.50–6.07	0.002
*Small–vessel occlusion based on TOAST classification*						
No	1.00					
Yes	0.17	0.10–0.29	0.001	0.58	0.21–1–60	0.3
*Undetermined etiology based on TOAST classification*						
No	1.00					
Yes	3.28	2.27–4.74	0.001	1.99	0.98–4.01	0.06
*TACI based on OCSP classification*						
No	1.00					
Yes	6.42	2.26–18–20	0.001	1.12	0.33–3.85	0.8
*LACI based on OCSP classification*						
No	1.00					
Yes	0.42	0.28–0.64	0.001	1.34	0.68–2.65	0.4
*Hemorrhagic transformation*						
No	1.00					
Yes	3.41	1.89–6.36	0.001	2.04	0.92–4.57	0.08
*Cortical stroke*						
No	1.00					
Yes	1.84	1.28–2.65	0.001	1.33	0.78–2.27	0.3
NIHSS at admission	1.18	1.13–1.22	0.001	1.14	1.09–1.19	0.001
*Hypertension*						
No	1.00					
Yes	1.76	1.18–2.63	0.005	1.15	0.63–2.12	0.6
*Atrial fibrillation*						
No	1.00					
Yes	2.72	1.85–4.00	0.001	1.59	0.80–3.15	0.2
*Hypercholesterolemia*						
No	1.00					
Yes	0.55	0.38–0.80	0.002	0.95	0.56–1.59	0.8
*Smoking*						
No	1.00					
Yes	0.58	0.38–0.88	0.01	1.34	0.72–2.49	0.3
*Hyponatremia*						
No	1.00					
Yes	2.02	1.02–4.02	0.04	1.74	0.72–4.24	0.2

* OR was also adjusted for the following variables: IS due to large artery atherosclerosis based on TOAST classification (*p* = 0.07 in univariate analysis) and hypernatremia (*p* = 0.08 in univariate analysis). OR = odds ratio; CI = confidence interval; IS = ischemic stroke; ICH: intracranial hemorrhage; TOAST: Trial of ORG 10,172 in Acute Stroke Treatment; OCSP: Oxfordshire Community Stroke Project; TACI: total anterior circulation infarction; LACI: lacunar circulation infarction; NIHSS: National Institute of Health Stroke Scale.

**Table 3:** Cox analysis, predictors of all-cause long-term mortality.

	OR	95% CI	*p*
Age	1.07	1.05–1.08	0.001
*Sex*			
Females	1.00		
Males	1.09	0.89–1.35	0.4
*Post-stroke seizures*			
No	1.00		
Yes	0.71	0.48–1.06	0.09
*Stroke subtypes*			
IS	1.00		
ICH	1.21	0.92–1.60	0.2
NIHSS at admission	1.07	1.05–1.08	0.001
*Pre-stroke mRS* > 2			
No	1.00		
Yes	1.65	1.30–2.09	0.001

OR = odds ratio; CI = confidence interval; IS = ischemic stroke; ICH: intracranial hemorrhage; NIHSS: National Institute of Health Stroke Scale; mRS = modified Rankin Scale.

**Table 4:** Type of seizure and long-term outcome.

	**SZR FA +** **(n = 15)**	**SZR −** **(n = 584)**	***p***
Disability, n (%)	9 (60)	379 (64.9)	0.7
Mortality, n (%)	8 (53.3)	436 (74.7)	0.06
	**SZR GTC +** **(n = 15)**	**SZR −** **(n = 584)**	***p***
Disability, n (%)	13 (86.7)	379 (64.9)	0.08
Mortality, n (%)	14 (93.3)	436 (74.7)	0.08
	**SZR FBTC +** **(n = 11)**	**SZR −** **(n = 584)**	***p***
Disability, n (%)	10 (90.9)	379 (64.9)	0.06
Mortality, n (%)	8 (72.7)	436 (74.7)	0.5
	**SZR FIA +** **(n = 10)**	**SZR −** **(n = 584)**	***p***
Disability, n (%)	8 (80)	379 (64.9)	0.3
Mortality, n (%)	6 (60)	436 (74.7)	0.2

SZR FA+: patients with focal aware seizures; SZR GTC+: patients with generalized tonic-clonic seizures; SZR FBTC+: patients with focal to bilateral tonic-clonic seizures; SZR FIA+: patients with focal impaired awareness seizures; SZR −: patients without post-stroke seizures.
